# 1,2,3,4,6-Penta-*O*-galloyl-β-D-glucose Inhibits CD44v3, a cancer stem cell marker, by regulating its transcription factor, in human pancreatic cancer cell line

**DOI:** 10.1080/19768354.2022.2152864

**Published:** 2022-12-03

**Authors:** Eun-Young Kim, Seong-Uk Lee, Yoon Hee Kim

**Affiliations:** Department of Food and Nutrition, Daegu University, Gyeongsan-si, Republic of Korea

**Keywords:** CD44s, CD44v3, PGG, cancer stem cell marker, ubiquitin

## Abstract

Inhibition of cluster of differentiation 44 (CD44), a pancreatic cancer stem cell (CSC) marker, is a potential treatment for pancreatic ductal adenocarcinoma (PDAC). In this study, we evaluated the effect of 1,2,3,4,6-penta-*O*-galloyl-β-D-glucose (PGG), a gallotannin contained in various medicinal plants, on CD44 standard (CD44s) and CD44 variant 3 (CD44v3) in Mia-PaCa-2, human pancreatic cancer cells and explored the underlying mechanisms. PGG showed cytotoxic effects and inhibited the proliferation of Mia-PaCa-2 cells. It also inhibited clonogenic activity, adhesion to fibronectin, and cell migration, which are characteristics of CSCs. PGG inhibited the expression of CD44s and CD44v3 by inducing the phosphorylation of p53 and suppressing NF-κB and Foxo3. Inhibition of Foxo3 induces CD44v3 ubiquitination. Indeed, PGG increased proteasome activity and promoted CD44v3 ubiquitination. PGG downregulated the CSC regulatory factors Nanog, Oct-4, and Sox-2, which act downstream of CD44v3 signaling. These data indicate that PGG may have therapeutic effects in pancreatic cancer mediated by inhibition of CSC markers.

## Introduction

Pancreatic cancer is associated with a poor prognosis, and its 5-year survival rate was 11% in USA in 2020 (Cancer.Net® 2022). More than 90% of pancreatic cancers are pancreatic ductal adenocarcinoma (PDAC) (Ishiwata et al. 2018). The poor survival, aggressive behavior, and resistance to chemotherapy of PDAC are partly attributed to the presence of cancer stem cells (CSCs) (Palamaris et al. 2021; Patil et al. 2021). CSCs, which have attracted attention as critical drivers of drug resistance and tumor recurrence (Patil et al. 2021), are a specific subset of neoplastic cells characterized by self-renewal capacity, clonal long-term repopulation potential, and the ability to divide asymmetrically, resulting in heterogeneous lineages of cancer cells (Chang and Pauklin 2021; Chen et al. 2021; Pook and Pauklin 2021). Pancreatic CSCs play a vital role in sustaining continuous tumor growth; they show higher chemoresistance as well as greater invasive and metastatic capacity than their differentiated cancer cell counterparts (Palamaris et al. 2021; Patil et al. 2021; Pook and Pauklin 2021). Studies have attempted to characterize and selectively target pancreatic CSCs (Pook and Pauklin 2021). CSC-specific cell surface markers have been extensively used for their isolation and characterization from various organs (Patil et al. 2021). Several CSC markers have been reported in PDAC including cluster of differentiation (CD) 44 (Ishiwata et al. 2018; Gzil et al. 2019; Palamaris et al. 2021; Patil et al. 2021; Pook and Pauklin 2021).

CD44 is a non-kinase transmembrane adhesion receptor that binds to hyaluronic acid (Gzil et al. 2019; Patil et al. 2021). CD44 is involved in processes such as cellular adhesion, angiogenesis, cytokines release, and muscle repair (Gzil et al. 2019). CD44 is encoded by 20 exons and goes through extensive alternative splicing to produce CD44 standard (CD44s) and CD44 variant (CD44v) forms (Zhao et al. 2016). Patients with CD44-positive pancreatic cancer have a shorter median survival (20.3 months) than those with CD44-negative disease (25.3 months) (Gzil et al. 2019). The CD44 ^+ ^CD24 ^+ ^ESA^+^ phenotype has a 100-fold higher tumor-initiating capacity than non-tumorigenic cancer cells, and these cells display distinctive stem cell features, including self-renewal and the ability to generate phenotypically diverse progeny (Patil et al. 2021). High levels of CD44 are associated with poor prognosis in cancer, and CD44v3 is related to cancer metastasis (Zhao et al. 2016). Zhao et al. identified a highly invasive, metastatic, mesenchymal-like subpopulation of PDAC cells with high levels of CD44s expression and stem cell-like properties (Zhao et al. 2016). The expression of CD44s and its isoforms in cancer cells suggests that CD44 plays a role in promoting tumorigenesis, and may thus be a molecular target for cancer therapy.

1,2,3,4,6-Penta-*O*-galloyl-β-D-glucose (PGG) is a gallotannin that is present at high concentrations in several medicinal herbs such as *Galla Rhois* (Kwon et al. 2013). PGG shows anticancer effects in various malignancies such as breast (Mendonca et al. 2021), colon (KawK et al. 2018), and pancreatic cancers (Bae et al. 2019). In previous work from our group, we showed that PGG has anticancer effects mediated by inhibition of CD44s in pancreatic tumors. However, the mechanism by which PGG suppresses the expression of CD44s remains unclear. In this study, we examined the molecular mechanisms underlying the effect of PGG on inhibiting the expression of CD44.

## Materials and methods

### Chemicals and reagents

For cell culture, fetal bovine serum (FBS), Dulbecco's modified Eagle's medium (DMEM), Antibiotic Antimycotic (AA) Solution, and 0.5% Trypsin-EDTA were purchased from Hyclone (Logan, UT, USA). CytoTox96® Non-Radioactive Cytotoxicity Assay and CellTiter96® Aqueous One Solution Assay of cell proliferation were purchased from Promega (Madison, WI, USA). PGG, crystal violet, and glutaraldehyde solution were bought from Sigma-Aldrich Co. (St. Louis, MO, USA). CytoSelect^TM^ 48-well Cell Adhesion Assay (Fibronectin, Colorimetric Kit) and CytoSelect^TM^ 24-well Cell Migration Assay (8 μm pore size, colorimetric format) kits were purchased from Cell Biolabs Inc. (San Diego, CA, USA). The Proteasome Activity Assay Kit was purchased from Abcam (Carlsbad, CA, USA). Immobilon® transfer membranes with a pore size of 0.45 μm were purchased from Millipore (Bedford, MA, USA). The antibodies used and commercial sources are as follows: anti-CD44 (Abcam), anti-Nanog, andti-Oct-4, anti-Sox-2, anti-Ubiquitin, anti-GAPDH (Santa Cruz Biotechnology, Dallas, TX, USA), anti-CD44v3 (R&D Systems, Minneapolis, MN, USA), anti-Foxo3 (Abcam), anti-NF-κB p65, anti-p-p53, anti-p53, anti-Lamin A/C, anti-mouse IgG HRP-linked antibody, anti-rabbit IgG HRP-linked antibody (Cell Signaling Technology, Danvers, MA, USA), and m-IgGk BP-HRP-linked antibody (Santa Cruz Biotechnology). The Pierce^TM^ Co-Immunoprecipitation Kit was purchased from Thermo Fisher Scientific (Bonn, Germany).

### Cell line and culture

The Mia-Paca-2, human pancreatic cancer cell line, was bought from the Korea Cell Line Bank (Seoul, Korea) and maintained as a monolayer culture in DMEM containing 10% FBS and 1% AA solution. Mia-Paca-2 cells were maintained at 37^○^C and 5% CO_2_ in the cell culture incubator and passaged every 2–3 days. The cells were treated with 0, 1, 2.5, 5, or 10 μM PGG for 48, 72 or 96 h.

### Cell proliferation and cytotoxicity assay

The cytotoxicity of PGG and the effect of PGG on cell proliferation were determined using the CellTiter96® Aqueous One Solution Assay of cell proliferation (Promega) and CytoTox96® Non-Radioactive Cytotoxicity Assay (Promega), respectively. Mia-Paca-2 cells were seeded on a 96-well plate at a density of 1 × 10^4^ cells/200 μL/well, and PGG was treated to each well at a range of concentration (0–10 μM) in DMEM containing 1% FBS. In the 0 μM PGG, cells were added with an equal volume of DMSO, and maintained at maximum concentration of not more than 0.001%v/v. After 48, 72, or 96 h, cytotoxicity and cell proliferation were examined according to the manufacturer’s instructions. The absorbance of each sample was read at 490 nm using a microplate reader (Sunrise; Tecan Austria, Salzburg, Austria).

### Colony formation assay

Cells (1,000) were seeded in 6-well plate, and 24 h later cells were exposed to PGG (0–10 μM) for 19 days; the medium was replaced by fresh medium containing PGG every 3 days. Then, cells were fixed with 6% glutaraldehyde and stained with 5% crystal violet. Visible colonies were counted and photographed.

### Adhesion assay

The effect of PGG on the adhesion of Mia-Paca-2 cells was assessed using the CytoSelect^TM^ 48-well Cell Adhesion Assay (Fibronectin, colorimetric kit) (Cell Biolabs Inc.) according to the manufacturer’s instructions. Briefly, cells were seeded on a 48-well plate at a density of 1 × 10^5^ cells/500 μL/well, and PGG was treated to each well at a range of concentration (0–10 μM) in DMEM containing 1% FBS and then incubated for 90 min at 37 ^○^C and 5% CO_2_ in a humidified incubator. Cells were fixed and stained. An extraction solution was added to each well, and the extraction solution was moved to a 96-well plate. The absorbance of the extraction solution was read at 560 nm in a microplate reader (Sunrise; Tecan Austria).

### Migration assay

Cell migration was assessed using the CytoSelect^TM^ 24-well Cell Migration Assay (8 μm pore size, colorimetric format kit) (Cell Biolabs Inc). Mia-Paca-2 cells were seeded in the inserts (8 μm pore size) in serum-free medium containing diverse concentrations of PGG (0–10 μM) at a density of 4 × 10^4^ cells/200 μL/well. The inserts were then placed in wells containing 500 μL of medium containing 10% FBS. Cells were incubated at 37^○^C for 48 h. After 48 h, the medium in the inserts was removed, and cells were carefully stained using the cell staining solution. Cells were harvested using extraction buffer, and absorbance was measured at 560 nm using a microplate reader (Sunrise; Tecan, Austria).

### Preparation of whole cell lysates

Mia-Paca-2 cells were lysed with lysis buffer according to previous reported study [13]. The lysates were collected by centrifugation at 12,000 × g for 15 min at 4°C and quantified using the Bio-Rad Protein Assay kit (Bio-Rad, Hercules, CA, USA). The lysates were stored at –80°C until needed.

### Nuclear and cytoplasmic extraction

Mia-PaCa-2 cells were seeded at a density of 1 × 10^6^ cells/3 mL/well in a 6-well plate, and PGG was treated to each well at a range of concentration (0–10 μM) in DMEM containing 1% FBS and then incubated for 48 h at 37°C and 5% CO_2_ in a humidified incubator. Then, nuclear and cytoplasmic fractions of cells were isolated using NE-PER^TM^ nuclear and cytoplasmic extraction reagents (Thermo Fisher Scientific, Bonn, Germany). The expression of NF-κB p65 was evaluated in the cytoplasmic and nuclear fractions by western blotting. Lamin A/C was used to adjust the relative values of western blot bands obtained from nuclear fractions.

### Western blot analysis

Total proteins were separated by 10% sodium dodecyl sulfate-polyacrylamide gel electrophoresis and transferred to Immobilon® transfer membranes with a pore size of 0.45 μm (Millipore). After blocking with 2.5% bovine serum albumin (Sigma) for 1 h, membranes were incubated with specific primary antibodies at 4°C overnight. Membranes were then washed with TBST20 solution and incubated with 2nd antibody conjugated to horseradish peroxidase (HRP) for 1 h. After washing, the protein bands were detected using an enhanced chemiluminescence reagent (Thermo Fisher Scientific) and the Fusion Solo system (Vilber, Lourmat, France). Densitometric measurements of band intensity were carried out using Image J free software (NIH, Bethesda, MA, USA).

### Proteasome activity assay

Mia-Paca-2 cells were seeded at a density of 1 × 10^5^ cells/3 mL/well in a 6-well plate, and PGG was treated to each well at a range of concentration (0–10 μM) in DMEM containing 1% FBS and then incubated for 72 h at 37°C and 5% CO_2_ in a humidified incubator. Proteasome activity in the samples was measured with a Proteasome Activity Assay Kit (Abcam) according to the manufacturer’s protocol. The fluorescence generated by the cleavage of 7-amino-4-methylcoumarin (AMC)-labeled substrate was established using a Tecan Infinite 200 Pro microtiter plate reader (Tecan, Mannedorf, Switzerland) at excitation 350 nm and emission 440 nm.

### Immunoprecipitation (IP)

Mia-Paca-2 cells were seeded at a density of 6 × 10^2^ cells/8 mL/well in a cell culture dish (90 mm × 15 mm), and PGG was added to each well at concentrations of 0, 2.5, 5, and 10 μM in DMEM containing 1% FBS and 1% AA solution. After 72 h of incubation, cells were lysed with lysis buffer. Total cell lysate protein (1 mg) was incubated with anti-CD44v3 antibody (20 μg/mL) overnight at 4°C. In addition, anti-CD44v3 antibody-mediated IP was performed followed by immunoblotting with anti-ubiquitin antibody.

### Statistical analysis

The experiment of this study was repeated at least three times, and GraphPad Prism Version 5.0 software (GraphPad, San Diego, CA, USA) was used for the statistical analysis. Data are presented as the mean ± S.E.M. of three independent experiments. Differences between the mean values of multiple groups were analyzed using one-way analysis of variance (ANOVA) with Dunnett’s test or two-way ANOVA with Bonferroni’s test. *P *< 0.05 was considered statistically significant.

## Results

### PGG inhibits the properties of cancer stem cells in PDAC

**Figure 1.**
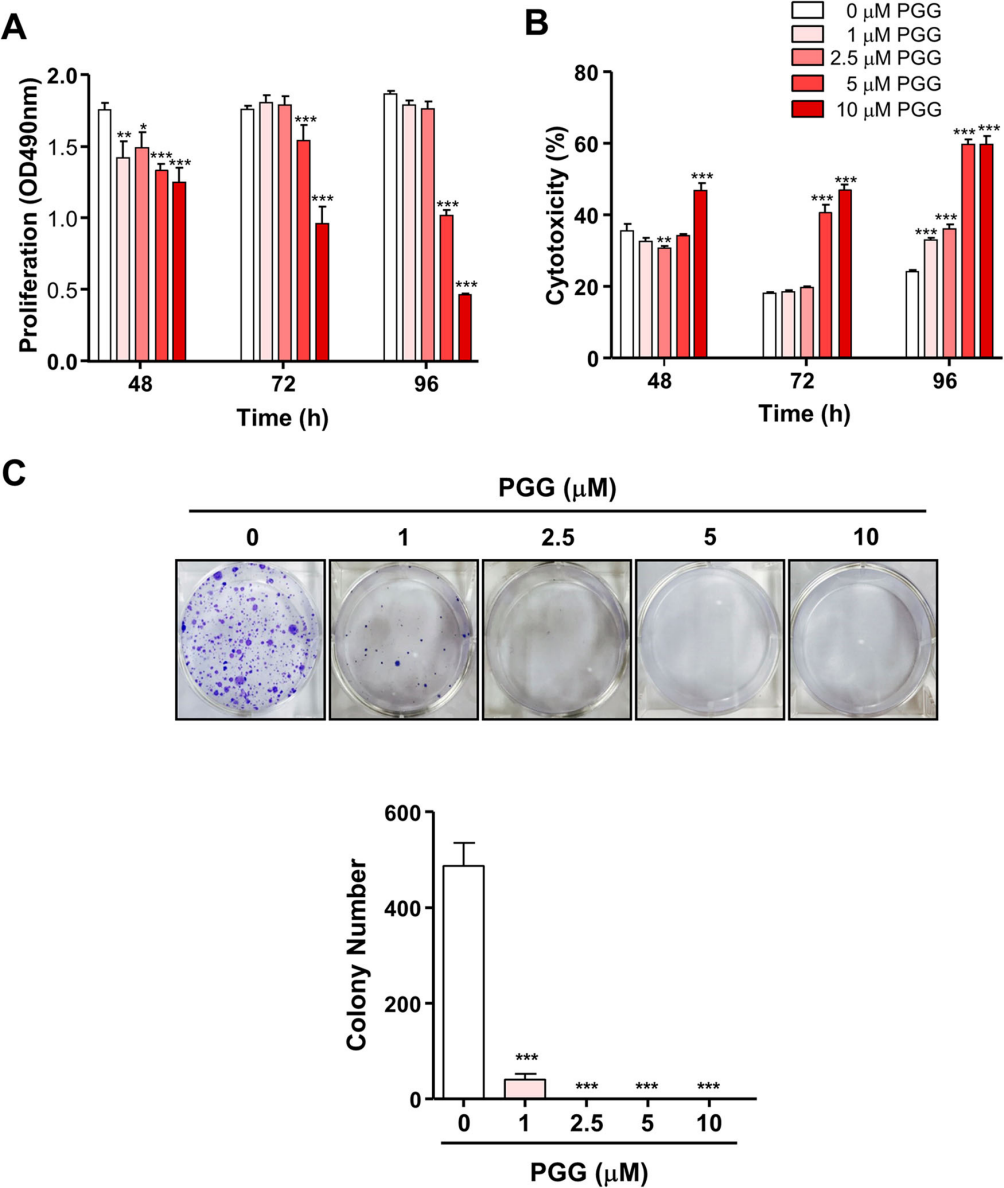
PGG inhibits Mia-Paca-2 cells proliferation, shows cytotoxic effect and inhibits colony formation of Mia-Paca-2 cells. Cells were treated with indicated the concentrations of PGG for 48, 72, or 96 h. Proliferation (A) and cytotoxicity (B) were determined as indicated in the main text. (C) Cells were treated with PGG at the indicated concentrations for 19 days. Colony formation was determined by crystal violet staining. Visible colonies were photographed and counted. Data are expressed as the mean ± SEM (n = 4). Statistical significance was based on the difference compared with 0 µM PGG by two-way ANOVA followed by the Bonferroni test (*P < 0.05, **P < 0.01, ***P < 0.001).

To evaluate the effect of PGG on cell growth in PDAC, Mia-Paca-2 cells were treated with different concentration of PGG for 48, 72, and 96 h. As shown in Figure 1A, at 48 h, the optical density (OD) was 1.76 ± 0.05 for 0 μM PGG, and it decreased to 1.25 ± 0.10 in response to 10 μM PGG. At 72 h, the OD of 0 and 10 μM PGG-treated Mia-Paca-2 cells was 1.76 ± 0.02 and 0.96 ± 0.12, respectively, whereas at 96 h, it was 1.87 ± 0.02 and 0.47 ± 0.01, respectively. PGG inhibited the proliferation of Mia-Paca-2 cells in a dose- and time-dependent manner (Figure 1A, *P < 0.05, **P < 0.01, ***P < 0.001). PGG-induced cytotoxicity in Mia-Paca-2 cells was observed in response to 10 μM PGG at 48 h (Figure 1B, ***P < 0.001) and increased in a time-dependent manner (Figure 1B). These data suggest that PGG has anticancer effects in PDACs.

**Figure 2.**
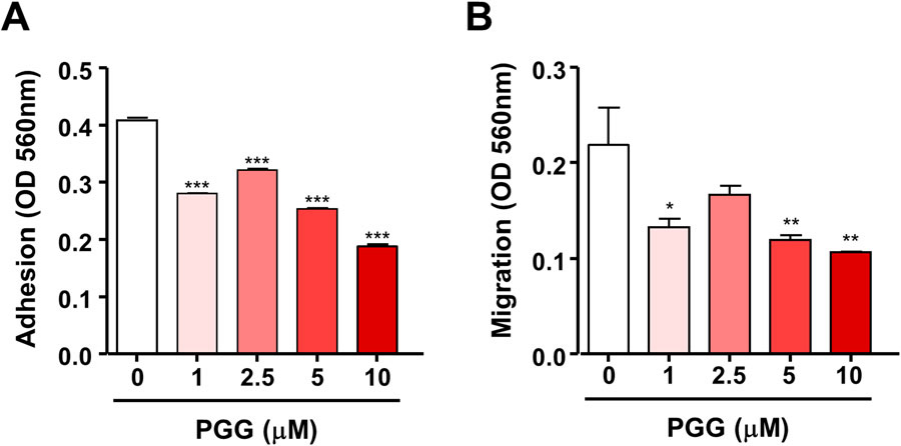
PGG suppresses cell adhesion and migration of Mia-Paca-2 cells. Cells were treated with the indicated concentrations of PGG. Adhesion (A) and migration (B) were determined as described in the main text. Data are expressed as the mean ± SEM (n = 3). Statistical significance was based on the difference compared with 0 µM PGG by one-way ANOVA followed by Dunnett’s test (*P < 0.05, **P < 0.01, ***P < 0.001).

Because PGG inhibited cell growth in PDAC and showed cytotoxic effects, we next examined the effect of PGG on the properties of CSCs such as self-renewal, migration, and adhesion. We used the colony formation assay, which evaluates the ability of single cells to become colonies *in vitro* (Franken et al. 2006), and evaluated the effect of PGG on colony formation. PGG inhibited the number of colonies stained with crystal violet staring at 1 μM (Figure 1C, ***P < 0.001). Next, we examined the effect of PGG on adhesion and migration, which are properties of CSCs, in Mia-PaCa-2 cells. Exposure to PGG inhibited adhesion of Mia-Paca-2 cells to fibronectin, an abundant extracellular matrix protein (To and Midwood 2011), starting at 1 μM PGG (Figure 2A, *** *P *< 0.001). The effect of PGG on the mobility of pancreatic cancer cells was examined using the Transwell assay. PGG decreased the migration of Mia-PaCa-2 cells at 1, 5, and 10 μM PGG (Figure 2B, *P < 0.5, **P < 0.01). These data suggest that PGG exerts anticancer effects by suppressing the properties of CSCs such as colony formation ability, adhesion, and migration.

### PGG downregulates the expression of the CSC markers CD44s and CD44v3

**Figure 3.**
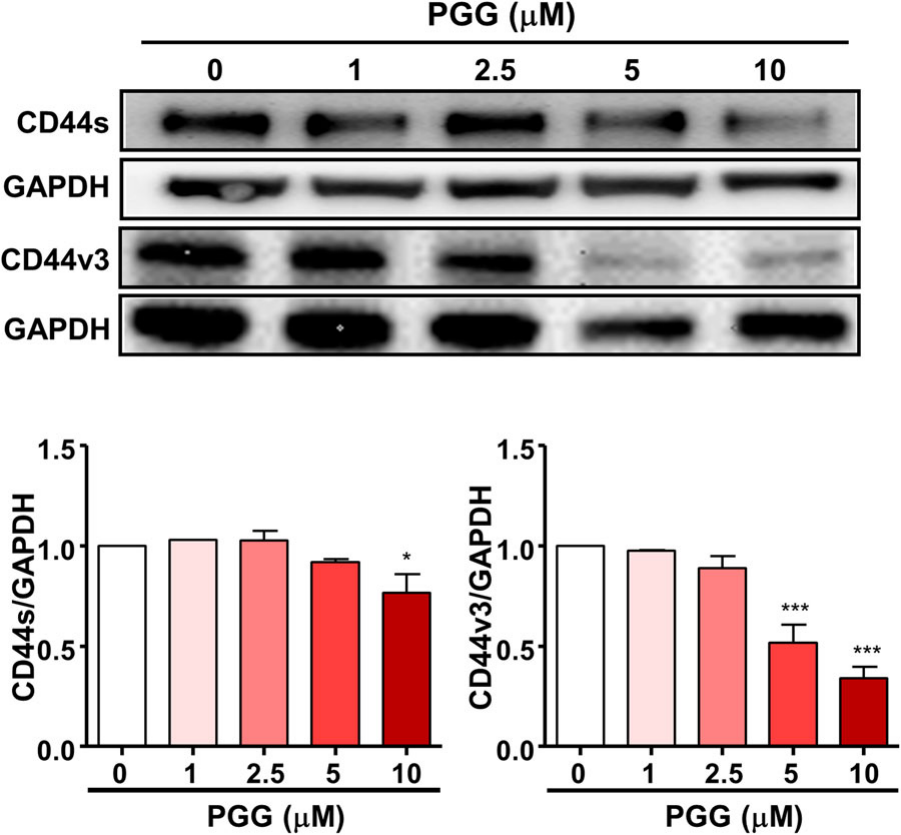
PGG inhibits the expression of CD44, a marker of pancreatic cancer stem cells. Cells were treated with the indicated concentrations of PGG for 72 h. Protein expression levels of CD44s and CD44v3 were determined. Relative values under western blot bands were calculated by dividing the density of CD44s and CD44v3 protein bands by the density of the GAPDH band. The data represent three repeated experiments and are expressed as the mean ± SEM (n = 3). Statistical significance was based on the difference compared with 0 µM PGG by one-way ANOVA followed by Dunnett’s test (*P < 0.05, ***P < 0.001).

Because PGG inhibited the properties of CSCs, we evaluated its effect on CD44, a marker of pancreatic CSCs. The results of western blotting showed that PGG downregulated the expression of CD44s and CD44v3 in Mia-PaCa-2 cells in a dose-dependent manner (Figure 3). These results are important because inhibition of CD44s and CD44v3 is sufficient to deplete the CSC phenotype in PDAC.

### PGG selectively regulates the expression of regulatory-transcription factors for CD44

**Figure 4.**
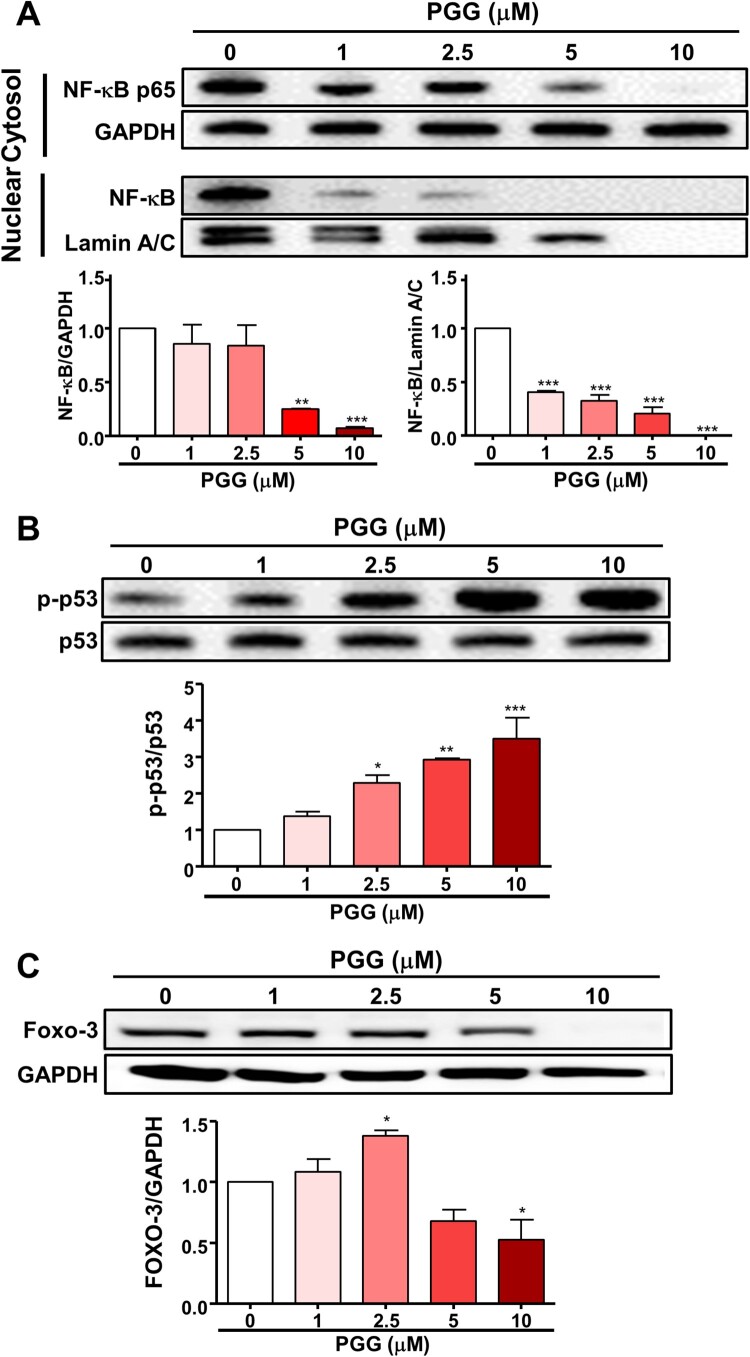
PGG selectively regulates the expression of transcription factors of CD44. Cells were treated with the indicated concentrations of PGG for 48 h. (A) Protein expression levels of NF-κB were assessed in nuclear and cytoplasmic fractions. Relative values under the western blot were calculated by dividing the density of the NF-κB p65 protein band by the density of the GAPDH band in the cytosolic fraction or the density of NF-κB p65 band by that of the Lamin A/C band in the nuclear fraction. (B) The relative value in the western blot was calculated by dividing the band density of the p-p53 protein by the density of the p53 band. (C) Relative values were calculated by dividing the intensity of the Foxo3 band by that of the GAPDH band. The data represent three repeated experiments and are expressed as the mean ± SEM (n = 3). Statistical significance was based on the difference compared with 0 µM PGG by one-way ANOVA followed by Dunnett’s test (*P < 0.05, **P < 0.01, ***P < 0.001).

Because PGG inhibited the expression of CD44s and CD44v3, we next focused on the effect of PGG on transcription factors that regulate CD44 expression, such as NF-κB, p53, and Foxo3, by western blotting. As shown in Figure 4A, PGG inhibited the expression of NF-κB p65 in the cytosol and nucleus. Lindenboim et al 2020. reported that Lamin A/C is included in the nuclear (NE) protein in the nuclear membrane, and NE is reduced by caspase-mediated cleaving when apoptosis occurs. PGG increased the phosphorylation of p53 in a dose-dependent manner (Figure 4B). Next, we examined the effect of PGG on Foxo3 expression in Mia-Paca-2 cells, which showed that PGG downregulated Foxo3 protein expression (Figure 4C). These data suggest that PGG suppresses the expression of CD44 by selectively regulating transcription factors for CD44.

### PGG promotes the ubiquitination of CD44v3

**Figure 5.**
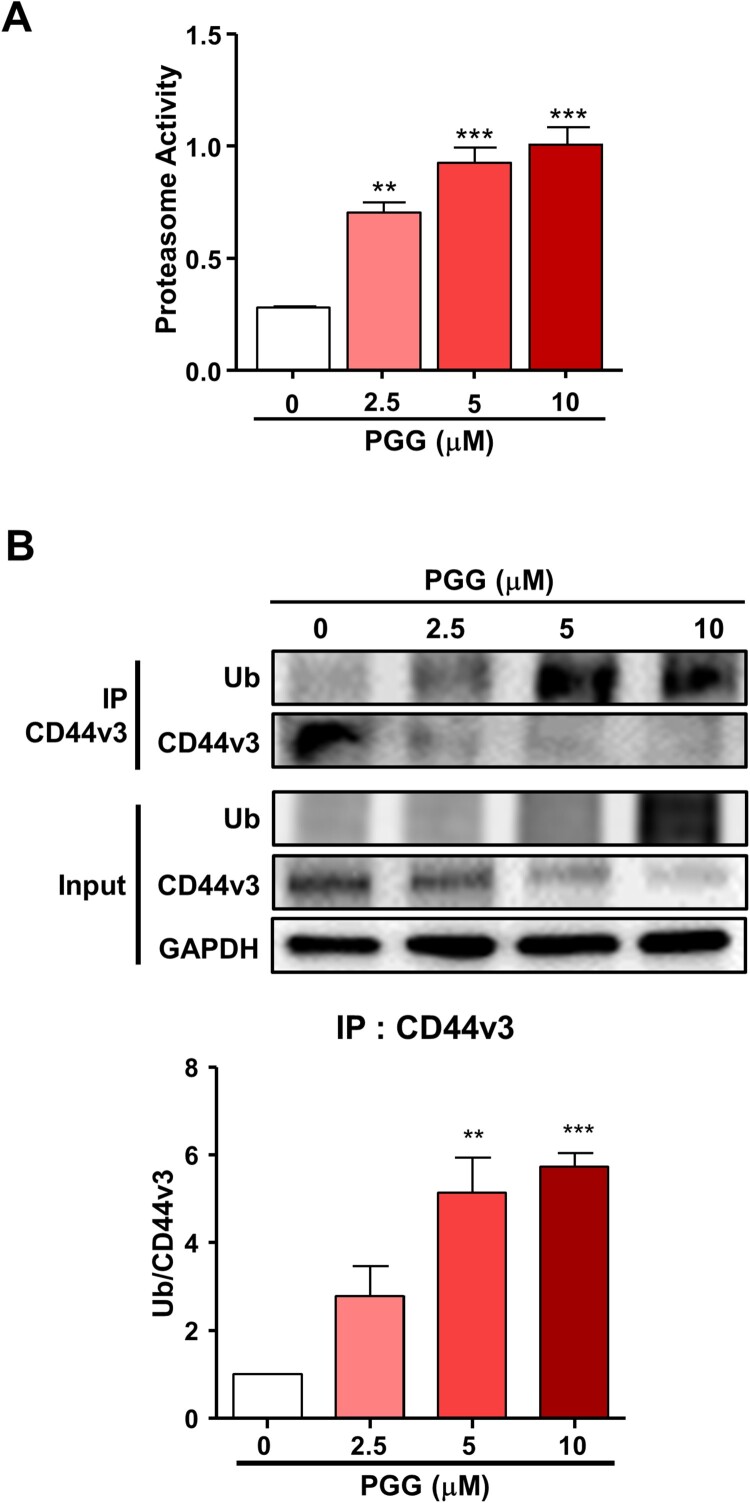
PGG activates the ubiquitin-proteasome system. Cells were treated with PGG at the indicated concentrations for 72 h. (A) Proteasome activity was determined as indicated in the main text. (B) Ubiquitination of CD44v3 was determined by IP. The data represent three repeated experiments and are expressed as the mean ± SEM (n = 3). Statistical significance was based on the difference compared with 0 µM PGG by one-way ANOVA followed by Dunnett’s test (*P < 0.05, **P < 0.01, ***P < 0.001).

The present data showing that PGG inhibited the expression of CD44s and CD44v3 and the reported by Kumazoe et al. that depletion of Foxo3 induces ubiquitination of CD44 (Kumazoe et al. 2016) led us to examine the effect of PGG on proteasome activity to determine whether the ubiquitin- proteasome system is involved in the modulation of CD44 expression. PGG increased proteasome activity starting at 2.5 μM PGG (Figure 5A, **P < 0.01, ***P < 0.001). To determine whether PGG induces CD44 ubiquitination, we performed an IP assay with anti-CD44v3 in PGG-treated Mia-Paca 2 cells. The results showed PGG induced CD44v3 ubiquitination (Figure 5B). These data indicate that PGG suppresses CD44v3 expression by promoting its degradation via the ubiquitin-proteasome system.

### PGG inhibits the expression of stem cell-specific transcription factors

**Figure 6.**
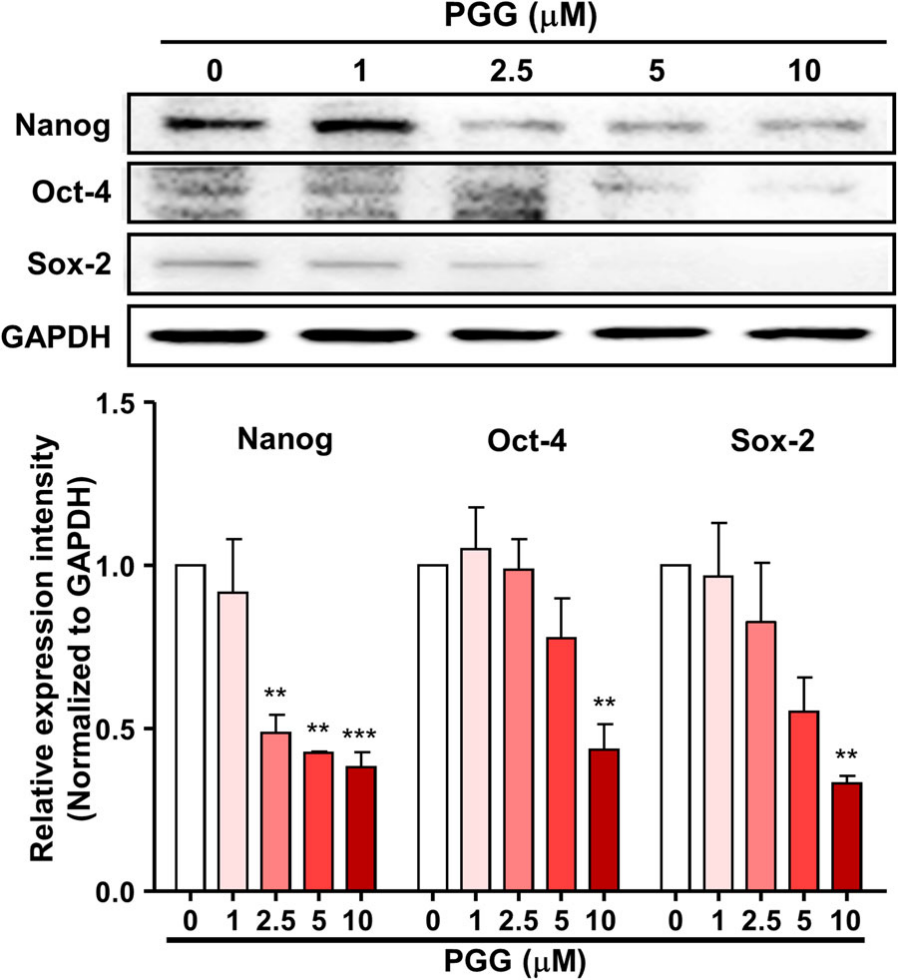
PGG suppresses the expression of CD44-related signaling pathway effectors. Mia-PaCa-2 cells were treated with different concentrations of PGG for 48 h and harvested. The expression of CD44-related proteins was analyzed by western blotting. Relative values under the western blot were calculated by dividing the density of the target protein band by that of the GAPDH band. The data represent three repeated experiments and are expressed as the mean ± SEM (n = 3). Statistical significance was based on the difference compared with 0 µM PGG by one-way ANOVA followed by Dunnett’s test (**P < 0.01, ***P < 0.001).

Because PGG downregulated CD44s and CD44v3, we next evaluated the effect of PGG on the expression of stem cell-specific transcription factors, which act downstream of CD44 and interact with each other. Treatment of Mia-PaCa-2 cells with PGG for 72 h downregulated the expression of Nanog, Oct-4, and Sox-2 (Figure 6). These data suggest that PGG inhibits CD44, thereby suppressing the activity of its signaling pathway.

## Discussion

The presence of pancreatic CSCs may underlie the poor survival, aggressive behavior, and resistance to chemotherapy of pancreatic cancer (Palamaris et al. 2021; Patil et al. 2021). Therefore, inhibition of CSCs may be a therapeutic strategy for the treatment of PDAC. Here, we examined the effect of PGG on the properties of CSCs and underlying molecular mechanisms. CSCs are characterized by unlimited proliferation, and self-renewal potential (Claudia et al. 2018; Barati et al. 2021; Tang et al. 2021). We thus used the colony formation assay, an *in vitro* cell survival assay based on the ability of single cells to grow into colonies (Franken et al. 2006). PGG inhibited colony formation in Mia-Paca-2 cells. Barati et al. suggested that metastasis and tumor recurrence are related to the presence of CSCs (Barati et al. 2021). Cell adhesion and migration, which are important factors affecting the metastasis of pancreatic cancer cells, are characteristic properties of CSCs (Gregory et al. 2009; Janiszewska et al. 2020). PGG suppressed cell adhesion and migration of Mia-Paca-2 cells. These data suggest that PGG exerts anticancer effects by suppressing the properties of CSCs such as colony formation ability, adhesion, and migration.

CD44 is an important surface marker for CSCs and thus, a potential target for the modulation of CSCs (Guo et al. 2021). PDAC with high expression of CD44 shows invasive, metastatic, and mesenchymal-like stem cell properties (Zhao et al. 2016). CD44 is involved in cancer progression, metastasis, and resistance to treatment (Guo et al. 2021), and the expression of CD44s is upregulated in human pancreatic cancer cell lines and in tumorous tissue samples from patients with pancreatic adenocarcinoma (Li et al. 2014; Wood. 2014). Blocking CD44 using an anti-CD44 monoclonal antibody decreases pancreatic cancer cell growth and invasion *in vitro* and inhibits tumor formation and recurrence *in vivo* (Li et al. 2014; Wood. 2014). In addition, CD44v3 is overexpressed in head and neck squamous cell carcinoma tissues and is associated with cell migration (Reategui et al. 2006). In breast cancer, the expression of CD44v3, v6, and v7/v8 is correlated with advanced stage disease (Gotte and Yip 2006). CD44v3 promotes CSC stemness in human head and neck squamous cell carcinoma (Bourguignon et al. 2012). Overexpression of CD44v3 is associated with transformation and progression in many cancer types (Todoroki et al. 2016). Because these studies suggest that CD44 is a potential therapeutic target, we examined the effect of PGG on CD44s and CD44v3 expression in Mia-PaCa-2 cells. In our data, PGG suppresses CD44s and CD44v3 expression in human pancreatic cancer cells. CD44 expression is correlated with histologic grade and poor prognosis (Hong et al. 2009). The expression of CD44v3 is correlated with metastasis in head and neck cancer as well as in colon cancer (Kuniyasu et al. 2002; Wang et al. 2007). These data are important because suppression of CD44s and CD44v3 is sufficient to deplete the CSC phenotype in PDAC.

Because PGG treatment suppressed the expression of CD44s and CD44v3 we next examined transcription factors that regulate CD44 expression, such as NF-κB, p53, and Foxo3. NF-κB is a positive regulator that increase CD44 promoter activity. NF-κB binds to the promoter of CD44 and is a key transacting factor that interacts with a conserved region upstream of the CD44 promoter (Chen et al. 2018). Pharmacological inhibition of NF-κB downregulates CD44 expression, thereby decreasing cancer cell proliferation and invasiveness (Smith et al. 2014). p53 is a negative regulator and inhibits CD44 promoter activity (Godar et al. 2008; Chen et al. 2018). Loss of functional p53 induces CD44 overexpression (Gzil et al. 2019). In this study, PGG inhibited NF-κB p65 and promoted the phosphorylation of p53. PGG inhibited the translocation of NF-κB p65 into the nucleus in lipopolysaccharide (LPS)-treated macrophages and UVB radiation-induced human dermal fibroblasts (Jang et al. 2013; Kim et al. 2015). Furthermore, PGG represses the translation of genes regulated by NF-κB (Mendonca et al. 2021). Consistently, we previously reported that PGG inhibits the translocation of NF-κB p65 into the nucleus of LPS-treated murine macrophages (Lee et al. 2017).

The transcription factor Foxo3 is expressed a high level in CD44-positive cells from patients with pancreatic cancer and in PDAC cell lines such as Pan-1, Mia-Paca-2, and BxPC-3 (Kumazoe et al. 2016). Foxo3 knockdown downregulates CD44 expression and inhibits colony formation (Kumazoe et al. 2016). In addition, inhibition of p53 through E3 ubiquitin-protein ligase MDM2 (MDM2) overexpression induced the expression of CD44 (Shayimu et al. 2021). In this study, PGG treatment suppressed the expression of CD44, and Foxo3. PGG activates p53 Ser15 phosphorylation in breast cancer and prostate cancer cell lines (Hu et al. 2008; Chai et al. 2010) and induces p53 expression in a colon cancer cell line (Kawk et al. 2018). These data that PGG treatment suppresses the expression of CD44 by selectively targeting its regulatory transcription factors. In this study, PGG treatment downregulated CD44s and CD44v3. Based on a report by Kumazoe et al. that depletion of Foxo3 induces CD44 ubiquitination (Kumazoe et al. 2016), we examined the effect of PGG on proteasome activity and ubiquitination of CD44. PGG increased proteasome activity and induced CD44 ubiquitination. Wei et al. reported that RNF128, a type I transmembrane protein that functions as ubiquitin ligases, directly interacts with CD44 and targets it for degradation by ubiquitination. In patients with melanoma, low RNF128 expression levels and high CD44 levels are associated with a poor prognosis (Wei et al. 2019). The membrane-associated RING-CH (MARCH), transmembrane E3 ligase, is downregulated in breast cancer tissues and interacts with CD44, a glycoprotein target subject to MARCH8-dependent lysosomal degradation in breast cancer (Chen et al. 2018). Therefore, degradation of CD44 through the ubiquitin-proteasome system is a potential therapeutic strategy for the treatment of cancer. Taken together, these data suggest that PGG inhibits the expression of CD44v3 by inducing its degradation by the ubiquitin-proteasome system.

The binding of ligand to CD44v3 promotes CSC stemness by triggering Nanog activation, which promotes the expression of pluripotent stem cell markers like Nanog, Sox-2, and Oct-4 (Guo et al. 2021). Binding of hyaluronan to CD44 promotes the recruitment and association between the cytoplasmic domain of CD44v3 and Nanog/Oct-4/Sox-2 (Chaudhry et al. 2021). Pluripotency-associated transcription factors such as Nanog, Oct-4, and Sox-2, which are overexpressed in CSCs, are key regulators of stemness in CSCs (Barati et al. 2021; Guo et al. 2021). Nanog, Sox-2, and Oct-4 promote tumor formation in ovarian cancer and prostate cancer (Lee et al. 2021; Robinson et al. 2021). The most commonly expressed CSC markers are CD44, aldehyde dehydrogenase, and CD133, which are involved in tumorigenesis, self-renewal, and therapy resistance, whereas NANOG, SOX-2, and OCT-4 are involved in metastasis and invasion (Preeti et al. 2021). Oct-4 is an important factor for pluripotency and is involved in cell differentiation, reprogramming and renewal (Gzil et al. 2019) Inhibition of both Oct-4 and Nanog decreases proliferation, migration, and invasion of pancreatic CSCs (Gzil et al. 2019). These markers are clinically important as potential therapeutic targets (Barati et al. 2021). Because PGG inhibited the expression of CD44, we hypothesized that it may inhibit factors in the CD44 signaling pathway, such as Nanog, Sox-2, and Oct-4. Indeed, PGG suppressed the expression of these pluripotency-associated transcription factors, suggesting that PGG inhibits CD44 and its downstream effectors.

## Conclusion

In this study, we showed that PGG inhibits the CSC properties of pancreatic cancer such as proliferation, colony formation, migration, and adhesion. PGG suppressed the expression of CD44, a marker of CSCs, by inducing its ubiquitination through the inhibition of Foxo3, as well as by selectively regulating the expression of transcription factors for CD44 such as NF-κB and p53. Inhibition of CD44 by PGG downregulated Nanog, Oct-4, and Sox-2, which are downstream effectors of CD44 signaling. Taken together, these data suggest that PGG has anticancer effects in pancreatic cancer.
